# Full circumpolar migration ensures evolutionary unity in the Emperor penguin

**DOI:** 10.1038/ncomms11842

**Published:** 2016-06-14

**Authors:** Robin Cristofari, Giorgio Bertorelle, André Ancel, Andrea Benazzo, Yvon Le Maho, Paul J. Ponganis, Nils Chr Stenseth, Phil N. Trathan, Jason D. Whittington, Enrico Zanetti, Daniel P. Zitterbart, Céline Le Bohec, Emiliano Trucchi

**Affiliations:** 1 Université de Strasbourg (UdS), Institut Pluridisciplinaire Hubert Curien, 23 rue Becquerel, 67087 Strasbourg Cedex 02, France; 2 Centre National de la Recherche Scientifique (CNRS), UMR 7178, 23 rue Becquerel, 67087 Strasbourg Cedex 02, France; 3 Centre Scientifique de Monaco (CSM), Département de Biologie Polaire, 8 Quai Antoine 1er, Monaco 98000, Principality of Monaco; 4 Laboratoire International Associé LIA-647 BioSensib, CSM-CNRS-UdS, 8 Quai Antoine 1er, Monaco 98000, Principality of Monaco; 5 Centre for Ecological and Evolutionary Synthesis (CEES), Department of Biosciences, University of Oslo, Postboks, Blindern, Oslo 1066, Norway; 6 Department of Life Sciences and Biotechnology, University of Ferrara, Via Borsari 46, Ferrara 44121, Italy; 7 Center for Marine Biotechnology and Biomedicine, Scripps Institution of Oceanography, University of California San Diego, 9500 Gilman Drive, La Jolla, California 92093, USA; 8 British Antarctic Survey, Natutal Environment Research Council, High Cross, Madingley Road, Cambridge CB3 0ET, UK; 9 Ocean Acoustics Laboratory, Alfred Wegener Institute, Helmholtz Centre for Polar and Marine Research, Am Alten Hafen 26, Bremerhaven 27568, Germany; 10 Applied Ocean Physics and Engineering Department, Woods Hole Oceanographic Institution, Woods Hole, Massachusetts 02543, USA; 11 Biophysics Laboratory, Department of Physics, University of Erlangen-Nuremberg, Henkestrasse 91, Erlangen 91054, Germany; 12 Department of Botany, and Biodiversity Research, University of Vienna, Rennweg 14, Vienna A-1030, Austria

## Abstract

Defining reliable demographic models is essential to understand the threats of ongoing environmental change. Yet, in the most remote and threatened areas, models are often based on the survey of a single population, assuming stationarity and independence in population responses. This is the case for the Emperor penguin *Aptenodytes forsteri*, a flagship Antarctic species that may be at high risk continent-wide before 2100. Here, using genome-wide data from the whole Antarctic continent, we reveal that this top-predator is organized as one single global population with a shared demography since the late Quaternary. We refute the view of the local population as a relevant demographic unit, and highlight that (i) robust extinction risk estimations are only possible by including dispersal rates and (ii) colony-scaled population size is rather indicative of local stochastic events, whereas the species' response to global environmental change is likely to follow a shared evolutionary trajectory.

## Abstract (web summary)

Delimiting populations is crucial for conserving threatened species. Using genome-wide data from the whole of Antarctica, Cristofari *et al.* show that Emperor penguins are organised into a single global population that have shared demography since the late Quarternary.

## Graphical abstract

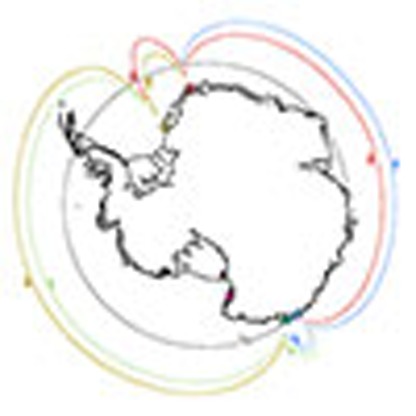

The rate of ongoing environmental change is now thought to exceed the rate at which most species^1^, including humans^2^, are able to adapt, with significant consequences for their resilience and for ecosystem sustainability. Consequently, an increasing number of studies seek to understand its impact on the world's ecosystems and to predict likely scenarios in response to climate projections^3^, either in order to set up more efficient conservation strategies, or as means to prompt urgent political action^4^. One of the main difficulties of this task lies in the fact that only a handful of species have been monitored for more than a few decades, and, in most cases, our knowledge of their demography is limited to local (that is, population-scale) and short-term (that is, generation-scale) responses^5^. Yet, in order to establish reliable projections, larger-scale population parameters must be integrated into demographic models. Recent developments in high-throughput sequencing allow the analysis of genome-wide and population-scale data and provide a genomic insight into the important demographic parameters that can be used to accurately predict species responses to global change^6^.

Extracting species-wide projections from time-series collected on a single population requires a precise understanding of how local events relate to species-scale demographic processes. Indeed, in several instances, it appeared that observations made on a single breeding population could not be extended to larger areas. The clear phenological shift in response to climate change observed in a Great tit *Parus major* population in Southern Britain^7^, for example, could not be observed under similar conditions in the Netherlands^8^. In the Chinook salmon *Oncorrhynchus tshawytscha*, survival rate in different populations was shown to be correlated with different sets of climate variables, thus preventing the definition of a single response model for the species^9^. In such cases, the confounding effect of local heterogeneity is often involved: for example, abiotic habitat characteristics^9^, biotic interactor communities^10^ or the dynamics of particular interspecific interactions^11^ can be locally heterogeneous, and lead to population-specific responses to climate change. Such observations present a challenge for the establishment of large-scale climate envelope models^12^, but also for the inference of species-wide demographic parameters from single-population surveys. Indeed, two independent assumptions need to be met: (i) that population dynamics are homogeneous in space (that is, that the focal population does not behave differently from the species as a whole), and (ii) most importantly, that populations are demographically independent, so that the observed local trends can be assumed parallel (and not complementary) to trends in other populations.

Indeed, robust extrapolation requires populations to be closed systems, in which coupling with other populations is minimal. In other words, we need to establish whether the observed local extinctions or fluctuations occur as a consequence of mortality peaks, or massive dispersal events—or a combination of both. Adult mortality has traditionally been proposed as the primary factor (through changes in resource availability and subsequent starvation^13^, or increased predation^14^), and several models have been built on that basis^15^,^16^. Yet, these models mostly rely on the explicit assumption that movement among populations is negligible^15^, leaving adult and juvenile survival and breeding output as the sole factors driving population dynamics. This assumption, however, is not based on direct evidence, but is rather motivated by technical difficulties in discriminating emigration from mortality of tagged individuals^16^.

In a recent review, Chown *et al.*^4^ reported that despite the pristine appearance of Antarctica, its species and ecosystems are also under considerable threat. The Emperor penguin, the only winter-breeding top-predator species of the continent, stands at the forefront of the impacts of climate warming^17^, and recent projections, based on the demographic trend observed at one colony in Adélie Land in Eastern Antarctica, suggest that it may be facing high extinction risk within the next 100 years^15^. Emperor penguins breed nearly exclusively on sea ice: this unstable habitat makes the species immediately sensitive to local environmental changes. One of the northernmost colonies, located on the Antarctic Peninsula, vanished during the last few decades, as sea ice retreated in that area^18^. Other colonies underwent a dramatic drop in breeding success and population size shortly after a modification in local sea ice topology^19^. The colony breeding on the tongue of the Mertz glacier, for instance, disappeared after the 2010 calving of that glacier^20^, and the following changes in local ice movements also had catastrophic consequences on the nearby Pointe Géologie colony, where the number of fledged chicks dropped from ∼2,500 in 2010 to ∼100 in 2014 (field observation). All of these events have in common an identified proximal cause, usually linked to modifications in the local sea ice landscape. Either increased sea ice forced adults to make longer trips to reach open waters for foraging (with subsequent breeding failure resulting from heightened energy expenditures)^19^, or on the contrary glacier calving destroyed the usual colony location^20^. On the other hand, recent empirical evidence increasingly points to an important effect of dispersal in the Emperor's response to habitat disruption^21^. The rapid recovery of the Emperor penguin population in Coulman Island confirms this assumption and excludes a peak in adult mortality followed by re-growth^19^. Recent satellite and ground surveys have also shown that whole Emperor penguin colonies are able to relocate with or without an identified cause^20^,^22^. Finally, biologging experiments have emphasized the outstanding distances regularly travelled by adult and juvenile Emperor penguins^23^. Here, we demonstrate that dispersal is a fundamental component of demography in this long-lived species, and that all the world's colonies behave as a single evolutionary unit sharing a common demographic history. We also propose that dispersal plays a central role in the species' adaptive response to environmental change at the continental scale. As such, migration among colonies needs to be incorporated into demographic models in order to achieve accurate projections.

## Results

### Genome-wide single-nucleotide polymorphism (SNP) typing

**Figure 1:**
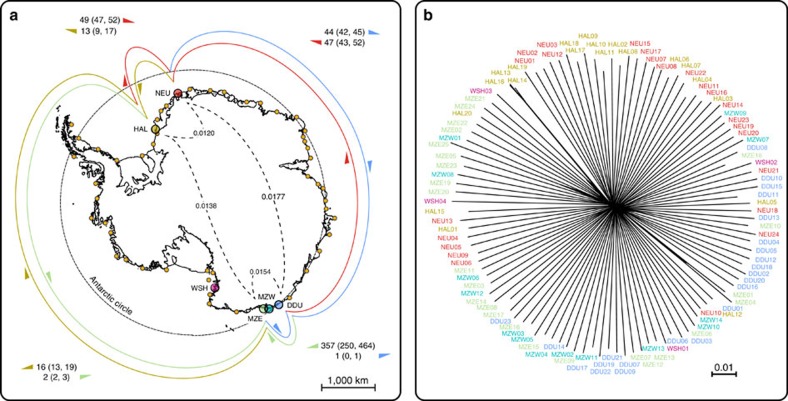
Drift and mixing concur to keep a high degree of worldwide homogeneity in the Emperor penguin. All currently known Emperor penguin colonies^20^,^35^ are represented by orange dots; sampled colonies by coloured circles. (**a**) Estimated pairwise migration rates (outer coloured links) and *Fst* (inner dashed links) between sampled colonies. Migrations as estimated from joint allele-frequency spectra. Colours refer to the receiving populations (DDU, Pointe Géologie near Dumont D'Urville station; HAL, Halley Bay; MZW, Mertz West; MZE, Mertz East; NEU, Neumayer; WSH, Cape Washington). Dashed circle: Antarctic polar circle (S66°33'). (**b**) Neighbour-net based on pairwise identity-by-state between all sampled individuals. Branch lengths represent pairwise Hamming distances. Colours correspond to colonies on map **a**.

We produced genome-wide restriction-site associated DNA sequencing (RAD-sequencing) data^24^, yielding a total of 59,037 highly confident polymorphic sites using a consensus-calling approach (Supplementary Fig. 1) for 110 individuals from 6 Emperor penguin colonies representing the whole species' range (Fig. 1a). To assess both fine- and large-scale processes, we sampled three colonies in a tight cluster around Adélie Land, in Eastern Antarctica: Eastern and Western Mertz colonies^20^ (‘MZE' and ‘MZW'), as well as the Pointe Géologie colony, near Dumont d'Urville research station (‘DDU'), all three within ∼300 km. Two colonies were sampled in the Weddell Sea area, across the continent: Atka Bay colony, near Neumayer research station (‘NEU'), ∼6,500 km away from Adélie Land, and Halley Bay colony (‘HAL'), ∼700 km further. Finally, one colony was sampled from the Ross Sea area (Cape Washington, ‘WSH'), ∼1,700 km from Adélie Land and ∼8,000 km from HAL.

### A fully panmictic species

In striking contrast both with its fragmented geographical distribution and with our current knowledge about other marine predators^25^,^26^,^27^, the Emperor penguin exhibits a remarkable degree of genetic homogeneity at the continent scale. Pairwise fixation index (*Fst*) values, calculated either as a function of allele frequency covariance between populations over variable sites, or from called genotypes using Reich's estimator^28^, are very low (Supplementary Table 1), and only ∼0.5% of total variance is explained by colony structure (as per analysis of molecular variance, or AMOVA). High genetic mixing is supported by all classical descriptors of genetic variation as estimated on our consensus SNP set. Hardy–Weinberg equilibrium holds across 466 out of 590 scaffolds with all colonies assessed together (out-of-equilibrium scaffolds all have less than 5 SNPs). The mean homozygosity across all individuals is low (F=0.051±0.094) and does not exhibit any inter-colony difference. The nucleotide diversity is low and highly similar across all colonies (*π*_DDU_=0.0026; *π*_MZE_=0.0023; *π*_MZW_=0.0023; *π*_NEU_=0.0023, *π*_HAL_=0.0023; *π*_WSH_=0.0026), going along with the expectation for long-lived high-investment species^29^, and despite very different colony census sizes. Finally, Tajima's *D*, as estimated for non-coding regions of the genome from down-sampled haplotypes, does not deviate from neutral expectations (*D*_DDU_=−0.87; *D*_MZE_=−0.66; *D*_MZW_=−0.578; *D*_NEU_=−0.64; *D*_HAL_=−0.68; *D*_WSH_=−0.28). A neighbour-net based on pairwise Hamming distances shows considerable admixture between areas: individuals are trending towards geographical sorting according to geographical location, but inter-individual variability is largely dominant (Fig. 1b). Overall, the variance explained by geographical structure is extremely low. The first principal component analysis (PCA) component of variance (Supplementary Fig. 2) shows a weak general pattern of isolation-by-distance along the coast. Mertz colonies stand on the one end, Pointe Géologie in the centre, and Atka Bay and HAL at the other end, yet Ross sea samples do not stand out, as would be expected in case of strong isolation-by-distance. However, this pattern remains extremely marginal: the variance explained by the first component barely amounts to 1.4% of the total variance, and colonies cannot be distinguished on the basis of PCA. Inference of population split topology based on allele frequency variation among populations also supports this view: neutral genetic differentiation from the ancestral population increases eastward from WSH to MZE, but with numerous migration events inferred between most colonies (Supplementary Fig. 3). Finally, in clustering analyses performed either on called genotypes or on genotype likelihoods, the preferred model consistently had *k*=1, that is, no inferred clusters in the data. The very limited genetic drift observed between colonies separated by several thousands of kilometres suggests intense gene flow in this flightless seabird along the coast of Antarctica.

### Long-term demographic reconstruction

**Figure 2:**
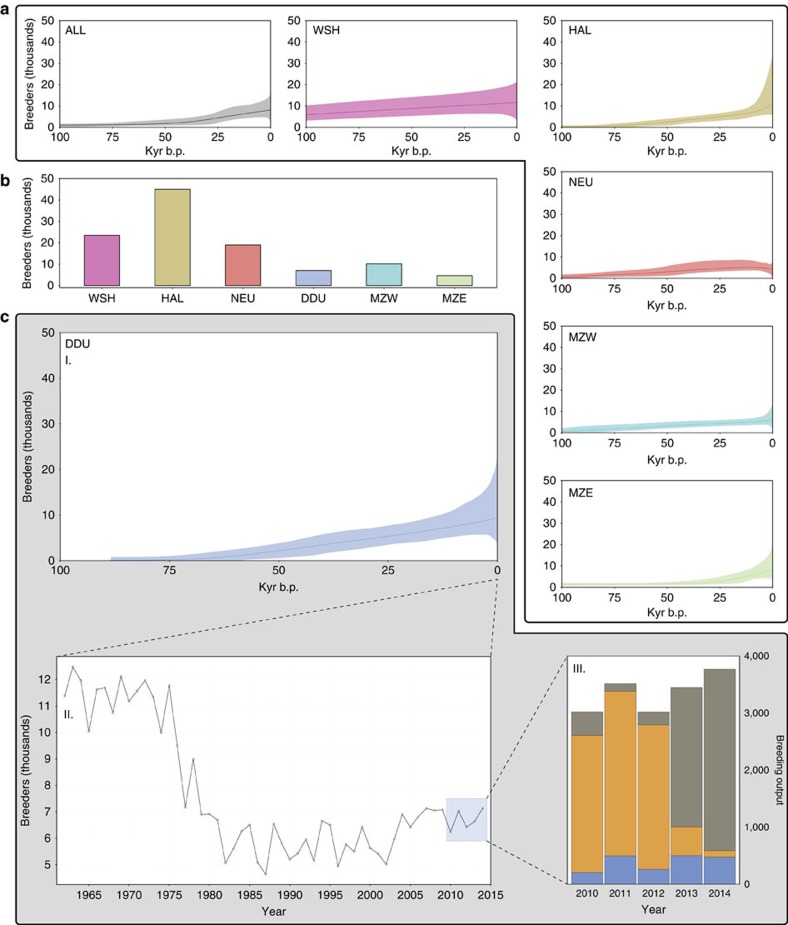
Demography is a matter of scale. (**a**) Demographic reconstructions (extended bayesian skyline plots), for all samples, and per-colony (ALL, all sampled colonies pooled together; DDU, Pointe Géologie near Dumont D'Urville station; HAL, Halley Bay; MZW, Mertz West; MZE, Mertz East; NEU, Neumayer; WSH, Cape Washington). Solid line: mean population size. Shaded area: 95% confidence interval. Blue area: Last Glacial Maximum (LGM). (**b**) Census size for the six analysed colonies (from Fretwell *et al.*^35^ and Ancel *et al.*^20^). (**c**) Three different demographic timescales for DDU colony: I. coalescent-scale (EBSP reconstruction), II. monitoring-scale (from Barbraud *et al.*^36^,^70^ and Le Bohec, personal communication, 2016), III. recent catastrophic breeding failure: blue, eggs lost during brooding; grey, chicks found dead; orange, successfully fledged chicks (from Barbraud *et al.* 70 and Le Bohec, personal communication, 2016).

The existence of a common, homogeneous gene pool for the entire species also implies that all present-day colonies share a common demographic history. To test this, we reconstructed past Emperor penguin population size changes in BEAST2 (ref. 30) under an extended Bayesian skyline plot model. In accordance with our expectation, reconstructions based either on a single colony or on haplotypes sampled randomly from the whole continent converge to the same estimate of effective population size and to the same demographic history (Fig. 2a). All reconstructions show a moderate increase in population size over the past 100,000 years (with some uncertainty as to the precise dating of the beginning of the expansion, because of the difficulty of precisely calibrating mutation rates in a multilocus approach), regardless of the very different present-day colony sizes (Fig. 2b). This trend is in accordance with the findings of Li *et al.*^31^ based on a single-genome pairwise sequentially Markovian coalescent approach. Emperor penguin population size does not appear to have been affected by the last glacial period. This is in stark contrast with the sudden post-glacial population expansion of the Emperor's sister species, the King penguin *A. patagonicus* from the sub-Antarctic region^32^, a difference probably explained by the contrasting breeding habitats and strategies of the two species, the King penguin being dependent on year-round ice-free breeding sites. The inferred long-term demographic stability also excludes past genetic fragmentation, which is known to reduce effective population size^33^. Thus, overall, the agreement of coalescent histories at both local and global sampling scales supports the idea that all extant colonies share the same genetic pool, and that the effects of neutral genetic drift between distant areas are durably counterbalanced by the intensity of gene flow.

### Importance of continent-wide dispersal

To quantitatively assess the importance of continental dispersal, we modelled the inter-colony migration rates required to generate such a level of genetic admixture. We first co-estimated effective population size and bidirectional migration rates between a subset of four colonies representing our whole sampling area by simulating genetic data under a continuous-time Markovian coalescent model against the observed two-dimensional allele frequency spectra, using a composite-likelihood approach^34^. Analysis was calibrated using a RADome mutation rate as estimated for the King penguin^32^ and a generation time of 16 years^15^,^16^. Effective population sizes all converge to an average of ∼4,000 (from 1,120 to 7,640) breeding individuals (detailed estimates in Supplementary Table 2 and Fig. 1a), in keeping with the observed median size of extant colonies^35^. Each colony is estimated to receive, on average, between 0.7% and ∼4.2% of its effective population size in migrants every generation. Thus, if we were to scale these results to present-day observed census size, a colony such as Pointe Géologie (DDU), with a count of ∼7,000 breeding adults, would exchange, on average, between ∼260 and ∼300 migrants (3.8–4.2%) per generation with the rest of the continent. Remarkably, strongly asymmetric gene flow pattern is inferred between DDU and MZE (Fig. 1a), with nearly all dispersal occurring towards MZE. It is relevant that the DDU colony has indeed gone through a strong population reduction in the recent past, and environment-induced adult mortality was suggested to be the main cause^13^,^15^,^36^. However, our results point towards a very central role of dispersal, as direct human disturbance (for example, effect of flipper-banding^37^) on the DDU colony may have led to vastly increased emigration flow from DDU to MZE. We further validated these results using a haplotype-based multilocus Bayesian approach as implemented in Migrate-n^38^. Model ranking using Bayes factor model choice gives clear support for a full-migration model with very high gene flow. Estimated migration rates (*M*) and population sizes (*Θ*) are highly homogeneous: mean mutation-scaled (*μ*) migration rate *M*=*m*/*μ*=2,358±130, mutation-scaled effective sizes *Θ*_WSH_=0.0017, *Θ*_MZE_=0.0018, *Θ*_MZW_=0.0019, *Θ*_DDU_=0.0019, *Θ*_NEU_=0.0018, *Θ*_HAL_=0.0019. Considering a conservative range of *μ* for our subset of loci^32^, we can estimate that each colony receives, on average, from 5.40% (±0.22) to 10.00% (±0.42) of its total effective size as migrants from other colonies at each generation. Although slightly higher, this estimate is of the same order of magnitude as the one derived from the joint allele frequency spectra.

## Discussion

It is important to note that the estimates we present here reflect the migration parameters averaged over many generations, and not the instantaneous dispersal rate (see Supplementary Note 1 for details). Therefore, neither population sizes nor migration rates should be interpreted as referring literally to the immediate state of extant colonies but rather to the average coalescence-based population size and migration rate over time. Dispersal itself is not necessarily a stationary process: it may be very heterogeneous in space and time (as was shown, for example, in the Little penguin *Eudyptula minor*^39^), while individual colonies may show positive or negative dispersal balance according to the current local habitat conditions and modifications.

It appears from our results that Emperor penguin colonies are largely open systems, in which demographic coupling with other colonies through dispersal is a fundamental mechanism. Recent observations suggest that this view may be extended to other seabird species^26^. For example, we have repeatedly resighted micro-tagged King penguins born on one colony in the Crozet Archipelago, at different colonies in the same archipelago, and at colonies in the Kerguelen Archipelago, 1,500 km away (Le Bohec, personel communication, 2016). As pointed out by Mayr^40^, ‘*a high dispersal ability is a necessity for occupants of temporary habitats'*. This necessity appears to be twofold. First, from a mechanistic point of view, habitat instability can force dispersal. Indeed, dispersal has been the immediate response for colonies facing habitat disturbance in the recent past^18^,^19^,^21^, and it can play an important role by adding flexibility to an otherwise rigid philopatric system, and allowing species to dynamically exploit the best breeding locations in rapidly changing polar environments. Second, high dispersal rates are expected to impose a high *migration load* on natural selection, and to counteract the effects of local adaptations^41^, thus allowing the species to preserve a gene pool that is adapted to a wider range of possible environmental conditions: it may thus itself be adaptive in highly unstable habitats. Yet, a direct consequence is that colony-level demographic events are of local rather than global significance. Population trends extracted from a single colony reflect the immediate quality of the focal location on a generation scale, rather than species-wide parameters (Fig. 2c). In this context, the colony is not a truly relevant demographic unit, but rather a transient aggregation of individuals at a particular point of time. To distinguish this structure from panmictic and metapopulation systems, we propose the term *synnome* for this combination of an exceptionally fragmented space and a very fluid gene pool (see full definition in Supplementary Note 1).

Although exceptional if compared with current knowledge about other marine predators, such as turtles^25^, marine mammals^27^ and most seabird species^26^, intense dispersal is expected to be a common evolutionary strategy in unstable high-latitude environments^26^, especially under ongoing climate warming. Importantly, most of the evidence accumulated up to now in seabirds has relied heavily on mitochondrial DNA^26^ (see also Supplementary Note 2 and Supplementary Fig. 5 for details), which is known to provide biased evidence due to its faster coalescence rate and non-recombining character^42^. SNP data can also provide evidence of more intense gene flow than mitochondrial DNA (as has been shown, for example, in the Black-footed albatross *Phœbastria nigripes*^43^). Therefore, several species that are currently treated as fragmented metapopulation systems, may in fact appear to be closer to the Emperor penguin's *synnome* structure once genome-wide data will be available.

Our findings highlight the importance of adopting a cross-disciplinary approach, integrating population genomics and behavioural ecology, to the study of population dynamics. Such an approach accounts for the temporally and spatially complex ecological processes that shape the structure of worldwide populations, and should help in the development of more robust and accurate demographic projections at the whole species level. These refined projections will then be more likely to allow estimation of the extent of threats to vulnerable species and identifying the proximal causes of their decline. As a single genetic population, Emperor penguins will respond to climate change through a unified evolutionary trajectory. This insight thus presents new challenges to our understanding of how current climate scenarios will impact upon the future of the most cold-adapted species in the world, an iconic bio-indicator of the delicate Antarctic ecosystem.

## Methods

### Sample collection and DNA extraction

Material was collected from six locations of East Antarctica, between 2004 and 2012: Cape Washington in the Ross Sea area (‘WSH'), Pointe Géologie (‘DDU') in Adélie Land, ‘MZE' and ‘MZW' in George V Land, Atka Bay (‘NEU') in Dronning Maud Land area and ‘HAL' in the Weddell Sea area. All procedures employed during field work were approved by national Ethical Committees, and authorizations to enter the breeding site and to collect samples from living or dead birds were delivered by the US Antarctic Conservation Act (permit no 2004-010), the French ‘Ministère de l'Enseignement Supérieur et de la Recherche' and the ‘Terres Australes et Antarctiques Françaises' (permit nos 2012-126, 2012-111 and 2012-117), the German Environmental Agency (Umweltbundesamt, permit no I 3.5–94003-3/295) and the British Antarctic Survey/University of Cambridge Animal Ethics Review Committee. On the DDU colony, blood samples were collected from 23 chicks before fledging. In WSH, MZE, MZW, NEU and HAL, muscle samples were collected from frozen chick carcasses collected around the colony (WSH: *N*=4, MZE: *N*=25, MZW: *N*=14, NEU: *N*=24, HAL: *N*=20). DNA was extracted using a spin-column protocol (Qiagen DNEasy Blood and Tissue kit, Qiagen) with minor modifications.

### Genome-wide SNP typing

SNP discovery and sequencing followed a single-digest RAD-sequencing protocol^24^. Genomic DNA was checked for degradation on a 1.5% agarose gel, and only samples with consistently high molecular weight were retained and quantified by fluorometry (Life technologies Qubit). A total of 110 samples were retained and sequenced in 5 distinct libraries. (i) ∼150 ng of genomic DNA per sample were digested with the restriction enzyme *Sbf*-I-HF (NEB); (ii) each sample was then ligated to a unique barcoded P1 adapter before pooling in a single library. The library was then sheared by sonication (7 cycles of 30 s ON—30 s OFF); (iii) sonicated libraries were concentrated to 25 μl by DNA capture on magnetic beads (beads solution/DNA=0.8:1), thus further reducing the carry-over of non-ligated P1 adapters, and the target size range fraction (350–650 bp) was then selected by automated gel electrophoresis (BluePippin); (iv) capture on magnetic beads using the same beads/DNA ratio (0.8:1) was then employed in all following purification steps (after blunt-end repairing, poly-A tailing, P2 adapter ligation and library enrichment by PCR). Magnetic beads were kept together with the library throughout the pre-PCR steps, and DNA was re-bound to the beads for purification using a PEG-8000-binding solution; (v) PCR amplification was performed in 8 × 12.5 μl aliquots pooled after the amplification in order to reduce amplification bias on few loci because of random drift. PCR was performed using NEB Phusion polymerase with the following cycles: 30 s denaturation at 98 °C, 18 cycles of amplification (10 min at 98 °C, 30 s at 65 °C and 30 s at 72 °C), and a final elongation of 5 min at 72 °C; (vi) the library was then quantified by a fluorimetry-based method (Life technologies Qubit), and molarity was checked on an Agilent Bioanalyzer chip (Invitrogen). A final volume of 20 μl for each library was submitted for paired-end sequencing on an Illumina HiSeq2000 sequencer (V3 chemistry, libraries 1–3) or HiSeq2500 (V4 chemistry, libraries 4–5), at the Norwegian Sequencing Centre, University of Oslo, spiked with 20% PhiX control library in order to reduce low-diversity bias.

### Sequence alignment and genotyping

Data processing was performed using the following workflow: (i) *Sequence demultiplexing.* Read quality assessment was made in FastQC (http://www.bioinformatics.babraham.ac.uk/projects/fastqc/). Samples were de-multiplexed according to in-line barcodes using Stacks v1.20 (ref. 44), low-quality reads were discarded and sequences trimmed to 95 bp. (ii) *Read mapping and filtering*. Demultiplexed fastq files were mapped to the published contigs of the Emperor penguin genome^45^ using Bowtie2 2.2.3 (ref. 46), with standard settings, allowing only end-to-end mapping. Resulting SAM files were filtered using Samtools 0.1.19 (ref. 47), PicardTools 1.113 (http://picard.sorceforge.net), and custom R and shell scripts in order to discard unpaired reads and full read pairs where at least one mate has a mapping quality score below 30. The resulting BAM files were then filtered for PCR and optical duplicates by comparing mapping position and CIGAR string, using Picard MarkDuplicates. This process also allowed to filter out most sequencing errors, as MarkDuplicates only retains the read with the highest average Phred score in each duplicate cluster. (iii) *SNP calling and genotyping*. Three independent algorithms were used for SNP and genotype calling, all of them built on a maximum-likelihood framework. (i) First-in-pair reads were exported as BAM files for the maximum-likelihood-based genotype caller built in the Stacks pipeline (ref_map.pl), with a maximum of five mismatches allowed between alleles at a single locus (both within and between individuals), correcting genotype calls using the information from the whole data set in the rxstacks programme. (ii) We used the same cleaned BAM files to simultaneously call both mismatch and indel polymorphisms in all samples using the GATK HaplotypeCaller pipeline^48^, with standard parameters, except for population heterozygosity which was set to 0.01. (iii) ANGSD 0.900 (ref. 49) was used to call SNPs and genotypes using a maximum-likelihood process with the Samtools mpileup/bcftools algorithm, using the complete sample allele frequency information as a prior. Genotype calls were only retained for comparison purposes with Stacks and GATK, however, downstream analysis was performed directly on raw genotype likelihoods, as this approach has been shown to be much more sensitive to weak structure than classical SNP-calling analysis^50^. Genotype calls from all three processes were formatted into VCF format using software-specific (Stacks and GATK) or custom scripts (ANGSD); further filtering and manipulation were done using VCFtools 0.1.12 (ref. 51). For analyses relying on SNP calls, an additional filtering step was performed by (i) extracting the list of consensus loci called as SNPs by all three independent algorithms, (ii) discarding genotypes with a coverage under 3 × for analyses sensitive to sequencing depth, (iii) removing loci genotyped in less than 75% of all individuals, and finally (iv) thinning down loci to keep only polymorphism distant of at least 1 kb, in order to minimize linkage between markers. (iv) *Sex-linked marker analysis*. To check for sex-specific dispersal or structure patterns, we repeated analyses for each sex separately, using either autosomal loci only or Z-linked loci only. We used the published genome annotations for bird gametologs^52^ to identify potentially Z-linked scaffolds. As females are heterogametic in birds, we expect non pseudo-autosomal Z-linked regions to be fully homozygous in females, but to be neutrally heterozygous in males. We therefore retained only scaffolds that had a clearly bimodal distribution of heterozygosity, with one mode at or close to 0 (a slight tolerance was allowed to account for misalignment, sequencing errors mistyped as SNPs due to low coverage, or the presence of a transposable elements with autosomal homologues). Fifteen scaffolds were ultimately retained. Scaffolds containing candidate Z-linked coding DNA sequences, but with no visible bimodal heterozygosity distribution, were excluded altogether from the analysis. Sex assignment was performed independently from each of the non-recombining Z scaffolds, and consistency of sex calls between scaffolds was checked manually.

### RAD data description

Each HiSeq sequencing lane yielded an ∼201,000,000 paired-end reads (±16,000) with a mean Phred score of 37, only part of which was dedicated to that project. After barcode demultiplexing and quality filtering, we retained an average 147,000,000 read pairs per library (±13,000,000). Concordant alignment rate was high (70.7±9.1%). However, after filtering, a large proportion of the reads was identified as duplicates and removed from further analysis. On average, 479,000 read pairs were retained per individual. We built an average 78,000 loci per individual (±17,000). Overlap between SNP calling methods was high: 111,686 SNPs were called by GATK (173,704 SNPs total), ANGSD (203,801 SNPs total) and Stacks (148,721 SNPs total; Supplementary Fig. 1). After filtering by coverage (minimum 3 ×) and missing data (minimum 75% representation), 59,037 highly confident SNPs (hereafter our ‘consensus SNP set', as opposed to the ‘full data set', which includes all sequencing data that passed our quality checks and aligned successfully to autosomal or pseudo-autosomal scaffolds) were retained for analysis, with a mean depth of 6.8 × . Of these, 582, spread across 15 scaffolds, were identified as unambiguously belonging to the non-recombining region of the Z chromosome.

### Pairwise FST, AMOVA and summary statistics

Pairwise fixation index was estimated by two independent methods. We calculated Reich's estimator^28^ on called genotypes of our consensus SNP set, using custom R scripts. Without calling genotypes, we calculated Reynolds' estimator^53^ on the full data set, taking into account uncertainty in site-allele-frequency, according to the method implemented in ngsTools^50^,^54^. Both methods were performed on a per-site basis and averaged over 1-kb non-overlapping sliding windows, as the ratio of the windowed sum of inter-population variance over the windowed sum of total variance. For the genotype-call-free method, only sites with a probability of being variant at least equal to 0.95 were included in the estimation. Mean homozygosity, nucleotide diversity and Tajima's *D* were calculated on a per-locus basis, using the consensus SNP set. To avoid possible biases due to low coverage in estimating nucleotide diversity and Tajima's *D*, we randomly sampled one haplotype for each individual, and performed calculations on this haploid subset. Calculations were made using *adegenet*^55^ and *pegas*^56^ packages, as well as custom R scripts. To quantify the proportion of variation at each organization level, AMOVA was performed on a 22,875 unlinked SNP data using Arlequin^57^, on a per-locus basis, with 1,000 permutations.

### Identity-by-state (IBS) and identity-by-descent (IBD)

Pairwise indicators of IBS and IBD were calculated in PLINK v1.9 (ref. 58) based on the consensus SNP set. Allelic distance for pairs of individuals is rather sensitive to uneven coverage (Supplementary Fig. 4, lower triangle), with lower-depth individuals appearing more similar to all other individuals than higher-depth ones. However, IBD inference, which takes into account the total genetic variance in the whole sample, appears more robust to coverage variation (Supplementary Fig. 4, upper triangle). Three individuals in the HAL colony (HAL13, HAL14 and HAL16, visible on Supplementary Fig. 4), as well as two in the MZE colony (MZE01 and MZE04, visible on Supplementary Fig. 4), appear markedly more related to each other than to the rest of the sample. Of these two clusters, only one individual was retained for inbreeding-sensitive analyses, such as PCA. Pairwise IBS (Hamming) distances were used to generate a neighbour-net using SplitsTree^59^.

### Principal component analysis

PCA was performed on called genotypes in the consensus SNP set using R library *adegenet*^55^, and on genotype likelihoods for the full data set using ngsCovar^54^. Analysis was performed either on all samples or keeping only one individual for each highly related cluster. When performed on all samples, analysis was mostly driven by the HAL relatedness cluster, with the first principal component of variance (PC) accounting for 2.84% of total variance. Interestingly, these outliers are also identified as a separate group in admixture analyses (see below), with high repeatability, although with only a slight gain in model fit compared with a single-population model (Evanno's Δ*K* over 10 replicates). However, discriminant analysis of principal components^60^ performed in *adegenet* on the consensus SNP set, and contrasting these outliers and the main sample group, showed that single-locus contributions were very low and evenly spread across the genome, thus excluding any strong directional selective phenomenon. After removal of all samples but one in each high-relatedness cluster, variation did not appear to be driven by outliers anymore.

### Population splits and migration topology

We used TreeMix^61^ to infer the topology of population splits and migrations from allele frequency variation among colonies. We produced RAD-sequencing data for the Emperor's sister species, the King penguin and processed it with the same protocol as for our Emperor penguin samples. We genotyped 15 King penguins from the Baie du Marin colony, on Crozet Archipelago, that were used as an outgroup for the analysis. We restricted our data set to ca 15,000 highly confident, unlinked SNPs shared between the two species. Three-population and four-population tests did not allow us to reject a tree-like topology in our data. Analysis was performed first using the King penguin outgroup, and bootstrapping over blocks of 500 SNPs, in order to assess the topology of relationships between our six Emperor penguin colonies. We used this topology to re-run TreeMix without the King penguin samples, but fixing the root according to our first results, in order to increase the resolution of the analysis (Supplementary Fig. 3).

### Clustering analysis

Clustering analyses were performed both on called genotypes, using fastStructure^62^, and on genotype likelihoods, using ngsAdmix^63^ and a stricter filtering was applied: as input in fastStructure we selected unlinked 6,825 SNPs with a maximum amount of missing data of 10%. In ngsAdmix, we filtered the data set to 3,020 high-confidence sites on the basis of probability of being variable, coverage and phred score. We tested a number of components ranging from 1 to 10, with three independent replicates. For fastStructure, we used a simple prior for *K* values ranging from 1 to 10, and a logistic prior for values from 1 to 3. Most likely number of components was chosen using fastStructure's chooseK.py script for fastStructure models and Evanno's Δ*K* method for ngsAdmix models.

### Coalescent-based analysis using BEAST2

An independent estimate of population size changes through time was performed in BEAST2 (ref. 30). We proceeded as above for locus selection and haplotype down-sampling. To remain agnostic as to population structure, we performed analysis on each colony separately, as well as on the whole data set, as the lack of strong population structure, evidenced by all other analyses, allowed us to sample haplotypes from the whole species without violating the model's assumptions. We used an extended bayesian skyline plot model^64^ in order to co-estimate present-day *Θ* and possible past fluctuations. We followed the protocol of Trucchi and colleagues^32^, but reduced the parameter space by defining only one site-model per locus class (five or six SNPs), using HKY models with empirical base frequencies, and allowing for rate variation in four discrete gamma categories. Kappa was linked across site models, according to our expectation for neutral variation, in order to alleviate computational load. All chains were run in duplicate to check for convergence and for a sufficient length to gather effective sample sizes (ESS)>200 for all parameters, which necessitated ca 1,000,000,000 steps on all models. We used the same estimates for mutation rate and generation time as in Migrate-n analyses. Reconstruction for WSH colony is much less precise because of the very small number of haplotypes (*N*=4) sampled per locus. However, present-day population size estimate converges with reconstructions based on the other colonies.

### Fastsimcoal2 analysis

Joint derived-allele frequency spectra were generated from the full data set in ANGSD 0.900 (ref. 49) for a subset of four colonies that encompass the whole continent (MZE, DDU, NEU and HAL). To polarize these spectra, we reconstructed the most likely ancestral base for all positions in the RADome. We selected 12 high-quality Emperor penguin samples covering the whole species' range, as well as 12 high-quality King penguin samples processed according to the same protocol. States at all positions were determined using GATK's Haplotype Caller pipeline^48^. We used BEDtools^65^ and VCFtools' vcf-consensus script^51^ to update the published Emperor penguin genome and establish a reference RADome for both the King penguin and the Emperor penguin, using only high-quality polymorphisms (phred-scale genotype quality ≥80), and including variable sites as ambiguity codes according to the International Union of Pure and Applied Chemistry (IUPAC) standard notation. We aligned this RADome to the Adélie penguin genome *Pygoscelis adeliae*^66^ using Bowtie2 (ref. 46), and extracted the corresponding regions. For each RAD locus, a maximum-likelihood unrooted tree was built in PhyML^67^, and maximum-likelihood ancestral sequence for crown-*Aptenodytes* was reconstructed using PAML^68^ and Lazarus (https://project-lazarus.googlecode.com/), using PhyML tree topology as a prior. Ancestral states were then used to determine the ancestral and derived alleles in the Emperor penguin.

Reconstruction of population sizes and migration events was performed through composite-likelihood maximization^34^, by simulating joint-spectra under a continuous-time Markovian coalescent model in fastsimcoal2.5.11 (ref. 69). For each run, we performed a maximum of 80 expectation-conditional maximisation (ECM) optimization cycles over the 12 retained parameters (population sizes and asymmetric migration rates between the four analysed colonies), each parameter optimization step requiring the generation of 100,000 simulated joint-spectra. We generated 50 non-parametric bootstrap replicates for each spectrum. For each bootstrap data set, and for the original data set, we ran 50 independent replicates, and retained the one with the highest log-likelihood. We assumed a mutation rate of 2.6e^−7^ subsitutions per site per generation as calculated for the King penguin^32^, and a generation time of 16 years^15^. Computation was performed on the high-performance Abel cluster at the University of Oslo, and required a total of ∼30,000 CPU hours.

We chose to restrict our analysis to a stepwise migration model for two main reasons. First, computational load increases rapidly with the number of estimated parameters, and higher complexity models could not be run with the necessary amount of replication. Second, as our model is not supposed to represent precisely the present-day state of the colonies, but rather parameters averaged over a long period of time, we do not expect the intensity of the migration flow to be much affected by the structure of the connectivity.

### Coalescent-based analysis using Migrate-n

All methods used above provide a robust framework for identifying groups even in weakly structured populations. However, all are, to some extent, functions of the covariance of allele frequencies between populations. In the hypothesis that our estimate of the frequency spectrum may be biased is some way, for example, by moderate sample size, we also performed a coalescent-based structure analysis using Migrate-n^38^. As opposed to frequency-spectrum-based approaches (see above), coalescent-based analysis relies on phased polymorphisms to infer population parameters. After verifying that the number of polymorphisms in each RAD locus followed a Poisson distribution of *λ* equal to the mean number of polymorphisms per locus, we selected 50 random loci comprising between five and six polymorphisms as an unbiased representation of the neutrally evolving part of the genome^32^. To correct for potential over-representation of sequences in case a heterozygous individual was mis-called as homozygous, we randomly sampled one allele only for each individual. For all colonies except WSH, we randomly picked a set of haplotypes in the population (16 in MZE, DDU, HAL and NEU, 14 in MZW, and 4 in WSH). We ran a cold chain and three heated chain of 50,000,000 generations, recording every 500 generations, with a 5,000,000-generation burn-in. We used a static heating scheme, raising the cold chain to a power of 1.5, 3 and 1e6, and proposing chain swapping every 100 steps. We used a uniform prior for population sizes (*Θ*), bounded between 0 and 0.1 (with a *δ* of 0.01), and for the migration rates (*M*), bounded at 4,000 with a *δ* of 400. Proper mixing under these conditions was ensured using the highest parametrization model (model 2, see below). We compared five models of increasing complexity: (i) a panmictic model, in which all colonies were gathered in one population, estimating only a general *Θ*, (ii) a full-matrix model, in which asymmetric migrations were allowed between all pairs of colonies, (iii) a stepwise model, in which asymmetric migrations were allowed, but only between neighbouring colonies, in a closed circle, (iv) a first meta-population model, in which the Ross Sea (WSH), George V and Adélie Lands (MZE, MZW and DDU) and the South Atlantic (NEU and HAL) were treated as three populations, with an asymmetric migrations between them, (v) a second meta-population model, in which the northernmost colonies (MZE, MZW, DDU and NEU) were separated from the Weddell Sea (HAL) and the Ross Sea (WSH). Models were ordered by log Bayes factor defined by Kass and Raftery as *ln*BF=2[*ln*(mL(model1))-*ln*(mL(model2))], with mL(model1) and mL(model2) being the marginal likelihoods for the two compared models, as calculated by thermodynamic integration. Under this model, each migration rate can also be expressed as a proportion of the receiving population's effective size as *m*=4·*M*·*μ.* Hence, the relative demographic importance of immigration for any given colony can also be expressed as Σ_i_=*n*(4·*Mi·μ*), with *n* being the total number of populations identified as gene sources for the focal population. To convert mutation-scaled estimates of *M* and *Θ*, we therefore need an estimate of *μ* (the mean number of substitutions·per site per·Myr) for the set of loci used in the analysis. As the number of sites in each locus is mainly due to the stochastic nature of the mutational process, and follows a Poisson distribution of parameter *λ* equal to the mean SNP density per locus (see above), we can consider that a single true mutation rate (*μ*) applies to the whole RADome. However, as our analysis is restricted to loci containing five to six SNPs, our estimates of *Θ* and *M* are not directly scaled by *μ*, but rather by posterior probabilities of *μ* conditional on the number of SNPs in each locus class. We used the class-specific mutation rate posterior probabilities as calculated for the King penguin^32^. As a conservative estimate, we used a range of rates fitting the 3-SNPs to 6-SNPs class loci. Using a generation time of 16 years^15^, these are *μ*_3snps_=1.14e^−6^ and *μ*_6snps_=2.16e^−6^. As these were estimated from a subset of 16 haplotypes, and we included 82 haplotypes in our analysis, we considered that variability was likely to be underestimated by Trucchi and colleagues^32^ compared with our sample. Thus, we did not consider higher estimates than those made for 6-SNPs loci.

### Data availability

Demultiplexed sequencing data are available from the Short Read Archive with the accession number SRP070516. Input files used in the main analyses are available from figshare.com (https://dx.doi.org/10.6084/m9.figshare.2949508.v1).

## Additional information

**How to cite this article**: Cristofari, R. *et al.* Full circumpolar migration ensures evolutionary unity in the Emperor penguin. *Nat. Commun.* 7:11842 doi: 10.1038/ncomms11842 (2016).

## Supplementary Material

Supplementary Information Supplementary Figures 1-5, Supplementary Tables 1-2, Supplementary Notes 1-2 and Supplementary References
