# Supplementary material for: Full circumpolar migration ensures evolutionary unity in the Emperor penguin
Source: Nat Commun. 2016 Jun 14;7:11842. doi: 10.1038/ncomms11842 (PMC4911614; doi:10.1038/ncomms11842)
Supplement: Supplementary Information — Supplementary Figures 1-5, Supplementary Tables 1-2, Supplementary Notes 1-2 and Supplementary References [file ncomms11842-s1.pdf]

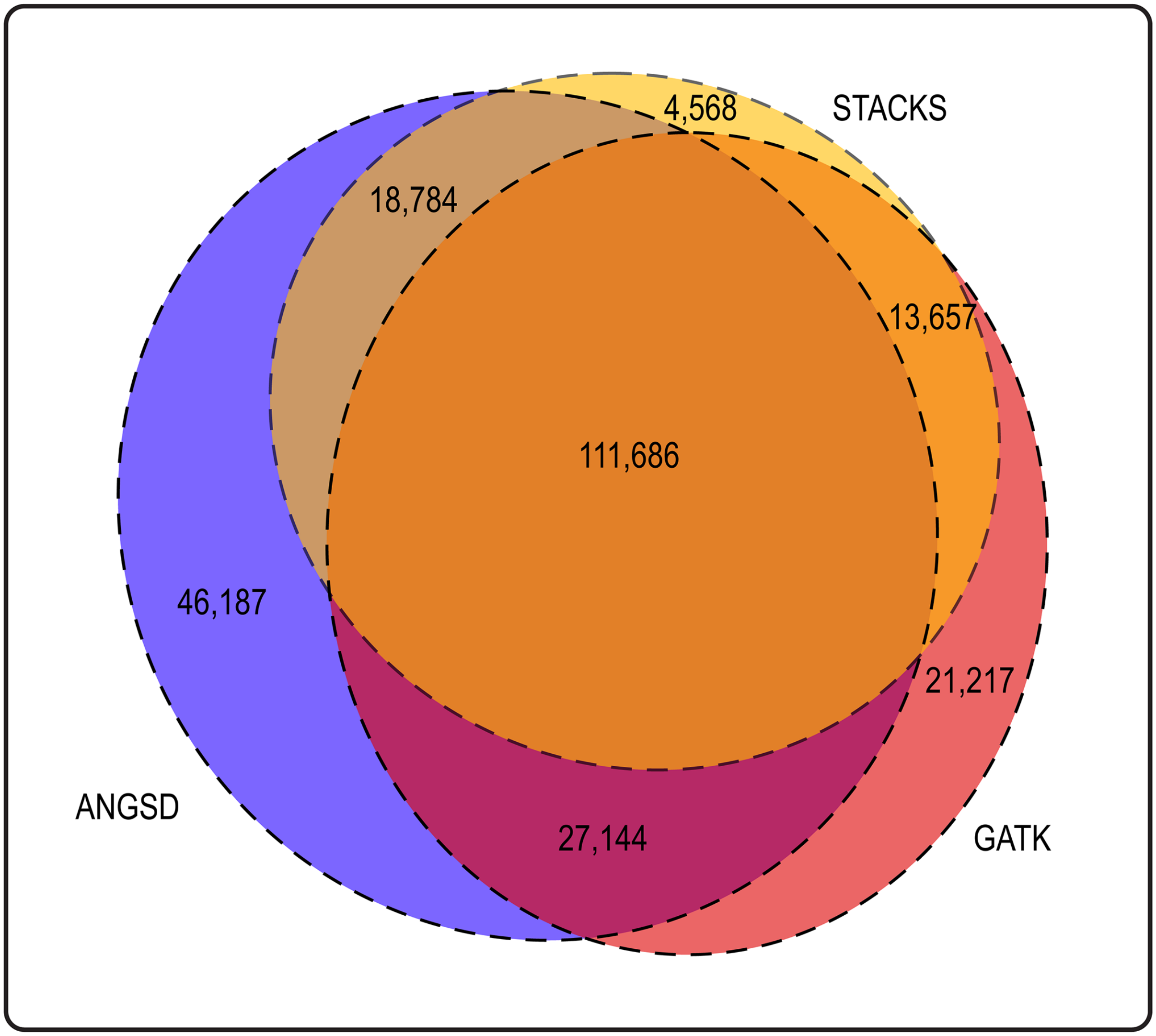

Supplementary Figure 1 | SNP calling. Venn diagram of SNP calls by three different algorithms: GATK, ANGSD with Samtools model, and Stacks (see Methods in main text). The 111,686 consensus SNPs were further filtered by coverage and missing data, yielding 59,037 highly confident SNPs retained for downstream analysis.

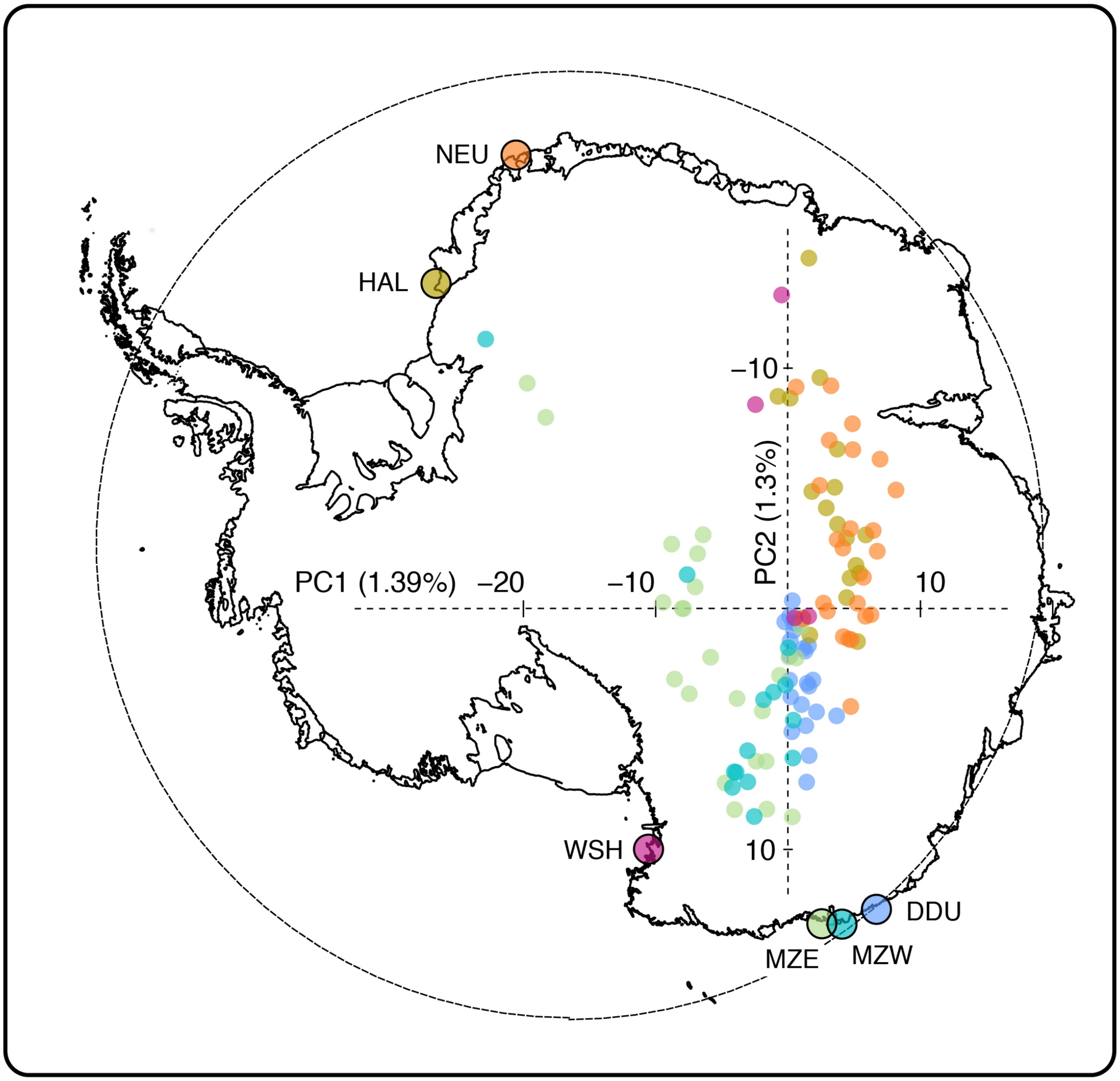

Supplementary Figure 2 | Principal component analysis. First two axes of a principal component analysis as performed from genotype likelihood data in ngsCovar (see Methods in main text). Point colours by colonies. The percentage of total variation explained by each component is indicated along the corresponding axis. Map overlay is only for visual reference.

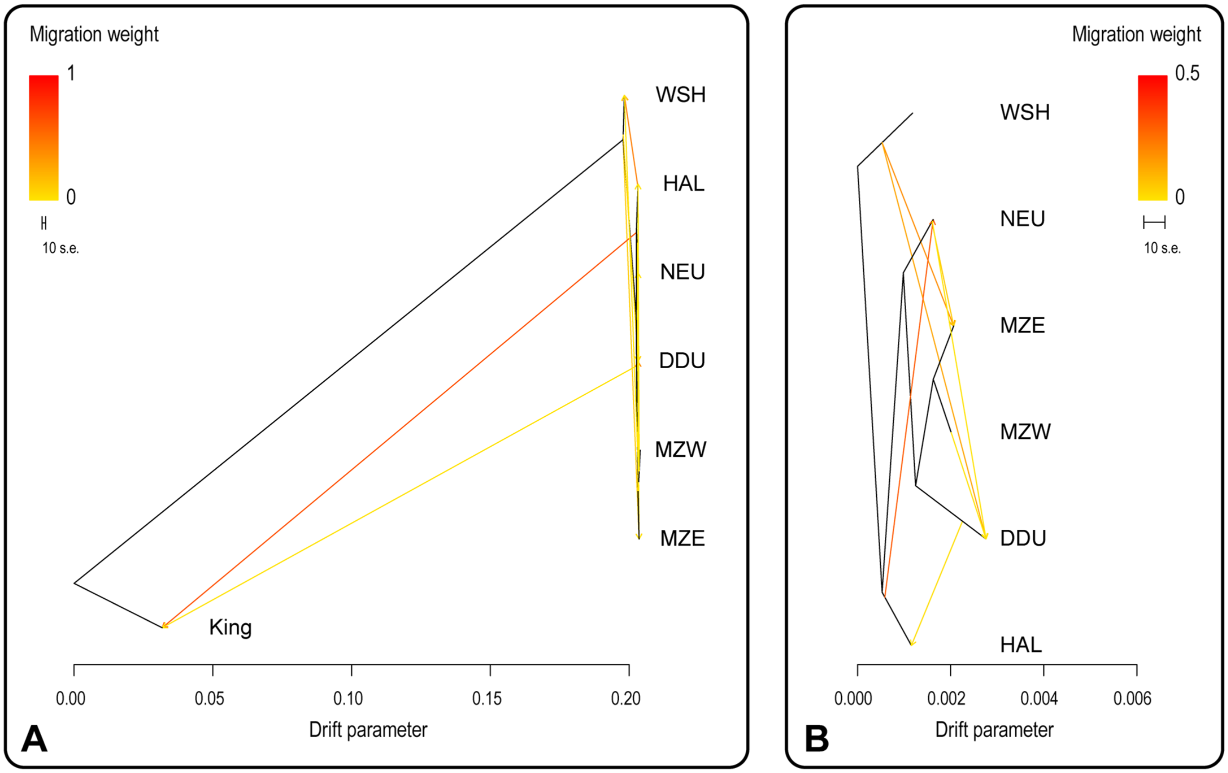

Supplementary Figure 3 | Inference of population splits. Population tree reconstruction based on the *TreeMix* model (see Methods in main text). (A) Including polymorphism data from the King penguin as an outgroup. (B) Without the King penguin and fixing the root as the same position as inferred in A.

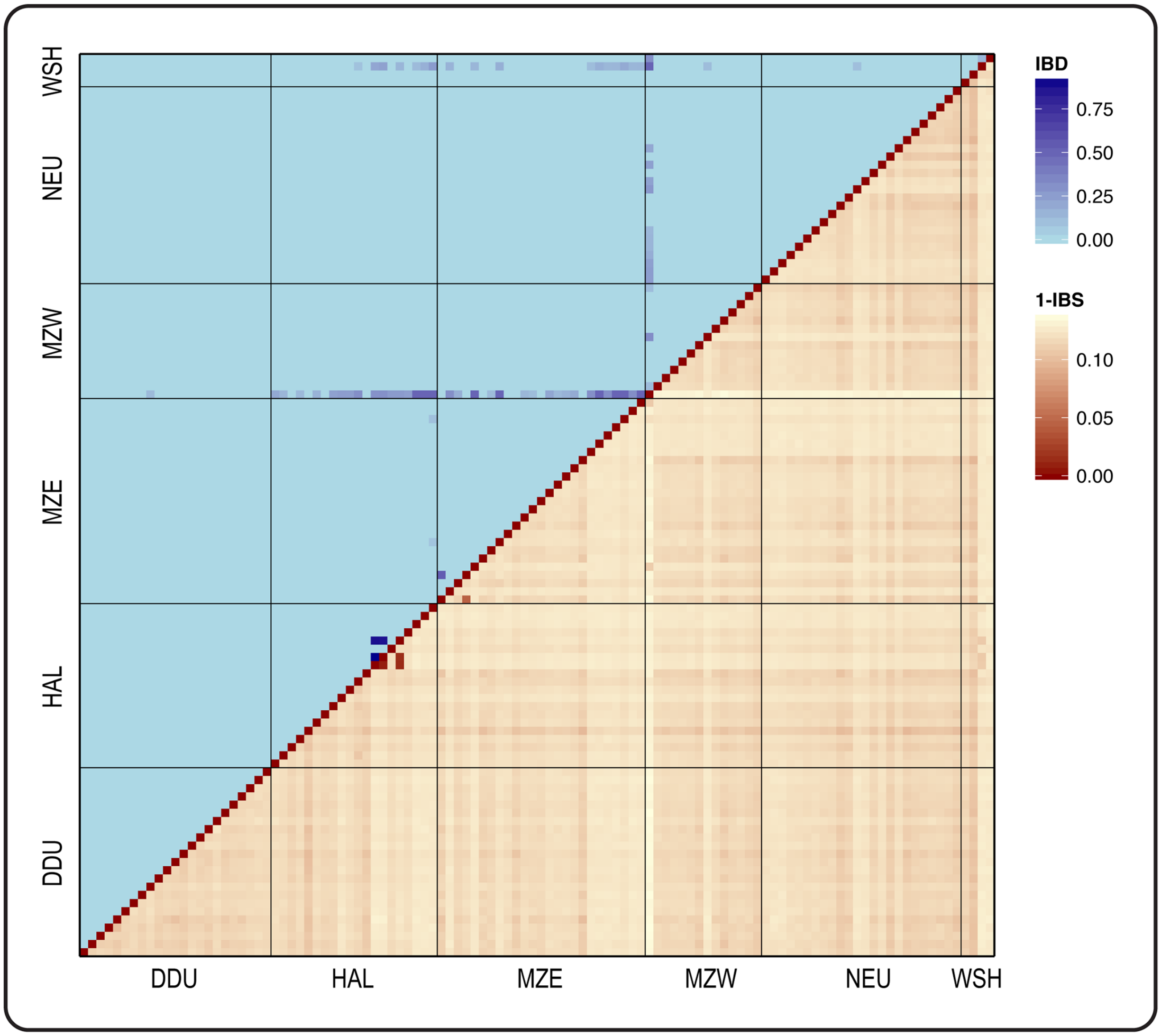

Supplementary Figure 4 | Pairwise genetic distance matrix. *Upper triangle:* identity-by-descent inference (IBD). *Lower triangle:* identity-by-state pairwise (IBS).

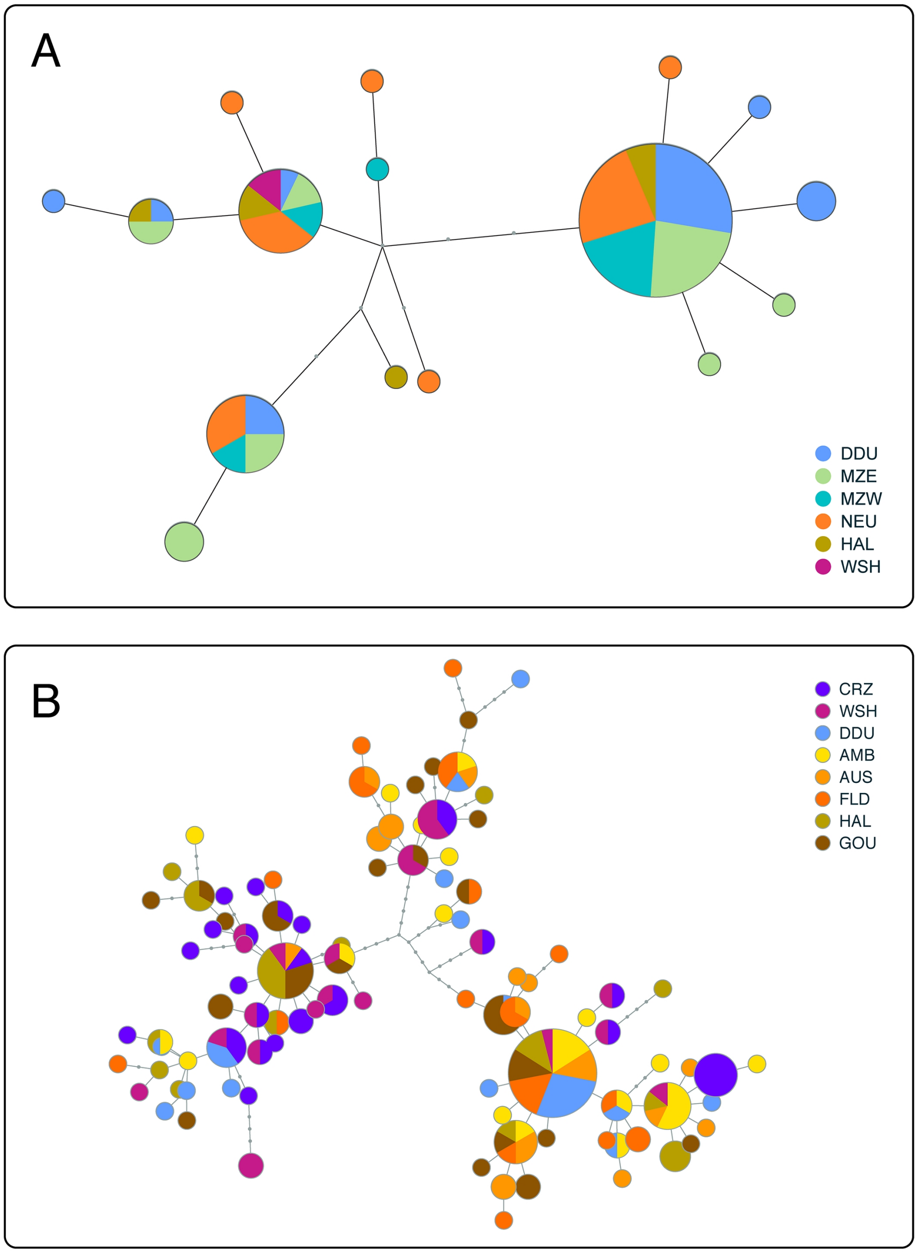

Supplementary Figure 5 | Haplotype network of mitochondrial sequences. Each dot represents a mutation. The area of the circles is proportional to the number of copies found in the sample. Coloured sectors indicate the geographical repartition of these haplotypes. (A) Cytochrome-*b* sequences produced for the present study. (B) Cytochrome-*b* and HVR sequences from Younger *et al.*[7], reanalysed for this study (Supplementary Note 2). CRZ: Cape Crozier, WSH: Cape Washington, DDU: Dumont d'Urville/Pointe Géologie, AMB: Amanda Bay, AUS: Auster, FLD: Fold Island, HAL: Halley Bay, GOU: Gould Bay.

Supplementary Table 1 | Pairwise Fst, according to Reich's estimator.

	DDU	HAL	MZE	MZW	NEU	WSH
DDU		0.0214	0.0154	0.0189	0.0177	0.0240
HAL	*0.0117*		0.0138	0.0177	0.0120	0.0137
MZE	*0.0107*	*0.0090*		0.0080	0.0112	0.0153
MZW	*0.0148*	*0.0115*	*0.0097*		0.0152	0.0202
NEU	*0.0120*	*0.0090*	*0.0084*	*0.0116*		0.0166
WSH	*0.0321*	*0.0274*	*0.0278*	*0.0302*	*0.0291*	

Upper triangle: mean value, lower triangle: standard deviation. DDU: Dumont d'Urville, HAL: Halley Bay, MZE: Mertz East, MZW: Mertz West, NEU: Neumayer, WSH: Cape Washington.

Supplementary Table 2 | (A) Migration rates and population sizes, (B) immigration rate per receiving population as ratio of population size, and (C) immigration rate per receiving population in effective breeders as estimated from the joint allele frequency spectrum.

A	mean	L95	U95
N_HAL	4040	3990	4080
N_NEU	3060	2920	3200
N_DDU	1120	1100	1150
N_MZE	7640	6150	9130
mHALNEU	0.0166	0.0153	0.0179
mNEUHAL	0.00323	0.0023	0.00416
mNEUDDU	0.0391	0.0376	0.0407
mDDUNEU	0.016	0.014	0.018
mDDUMZE	0.0416	0.0379	0.0452
mMZEDDU	0.000868	0.000372	0.00136
mMZEHAL	0.00397	0.00327	0.00466
mHALMZE	0.000413	0.000283	0.000544

B	mean	L95	U95
HAL	0.0072	0.00557	0.00882
NEU	0.0326	0.0293	0.0359
DDU	0.039968	0.037972	0.04206
MZE	0.042013	0.038183	0.045744

C	mean	L95	U95
HAL	29	22	36
NEU	132	117	146
DDU	161	152	172
MZE	170	152	187

Supplementary Note 1. Definitions.

Choice and definition of a « synnome ». For the lack of an appropriate term that would be currently in use, we chose to designate by the word *synnome* the particular colonial system that can be observed in the Emperor penguin, and possibly in other species. We derive this term from the Greek σύννομος, « *a common grazing of flocks* », used in particular for gathering flocks of birds (e.g. Aristophanes' *Aves*, v. 1756-7), and which was commonly used by extension for « *reunions* », « *gatherings* », and even « *kindred* ». These different meanings together convey the particularity of the observed structure: *one single, nearly homogeneous pool of individuals is distributed in a highly discrete way throughout its range. Local concentrations, or colonies, are highly consistent on the scale of a few generations, at which scale philopatry may be the norm. Yet, migration between these areas is high enough to maintain total homogeneity of the species' gene pool, so that, viewed on a micro-evolutionary time-scale, the only relevant unit is the species as a whole.*

Dispersal and migration. When considering the movements of individuals in a population system, an unfortunate complication often arises because of the convergent choice of the term « *migration* » to describe very different phenomena, in different conceptual frameworks. The first and most common sense of ***migration***, especially in an avian biology context, is the seasonal movements of groups of individuals between distinct breeding and overwintering grounds. Migratory species have evolved particular adaptations[1] that allow them to achieve well-timed departure and arrivals to track the most beneficial environments year-round. Such migratory patterns will not be examined in this work, mainly because it is not classically observable in our focal system (although the inter-breeding foraging trips of penguins may arguably be related to migratory behaviour[2]).

A simpler, one-way movement is the ***dispersal*** of individuals out of their original group. Dispersal was originally described by Howard[3] as « *the movement the animal makes from its point of origin to the place where it reproduces* ». From each individual's perspective, it is « *the greatest distance its genetic characteristics are transmitted, rather than the greatest distance the animal may have migrated or otherwise travelled away from the place it was conceived, hatched or born* ». Thus, genetically, a dispersal event is the « unit » of gene flow, with each dispersing individual bringing a set of alleles from one location to another.

The second sense of ***migration***, and the one we will use throughout this work, has been described by Dingle and Drake[4] in a biogeographical context as « *range expansions of faunas or individual species* », such as « *the northward extension of ranges following the retreat of glaciers at the end of the ice ages* ». More specifically, in a population genetics context, the (mutation-scaled) migration parameter *M* has been defined by the same authors as « *the exchange of genes among populations by whatever means, including but not limited to migration as we consider it here* »[4]. It is used in that sense in the coalescent framework, in particular by Beerli and colleagues[5,6]. Thus, in that context, migration is distinguished from dispersal by its larger scale: whereas dispersal is an individual- and generation-centred phenomenon that may be observed directly, migration is a time-averaged, population-centred event that is only detectable through indirect methods, such as gene flow reconstruction.

Supplementary Note 2. Re-analysis of mitochondrial DNA data published in Younger *et al.*[7] and comparison with novel data.

A recent study by Younger and colleagues[7] focused on Emperor penguin mitochondrial DNA population structure. Their conclusion was that colonies from the Ross Sea area are significantly isolated from the rest of the continent, and had a different demographic history. Our genome-wide SNP data does not support this view. However, our low sampling size in the Ross Sea region does not permit any definitive conclusion. In order to assess how far this result could be reproduced from mtDNA alone, we sequenced a 792 bp fragment of mitochondrial cytochrome-*b* gene and a 414 bp-long HVR fragment for our RAD samples, following the same protocol as Younger *et al.*[7]. Primers were the following: Cyt-b forward primer 5'-GCCCCAAACCTCCGAAAATCCCA-3' and reverse primer 5'-TGTGGAGGAGGGGATTAGG-3'; HVR forward primer 5'-GGAACCTCCCAAAGAGTACCA-3' and reverse primer 5'-CCAACCAGATGTATCGGTGA-3'. PCR conditions were thus: for Cyt-b, 5' denaturation at 94°C, 35 cycles of amplification (30" denaturation at 94°C, 30" annealing at 57°C, and 1' elongation at 72°C), and 5' final elongation at 72°C; for HVR, 5' denaturation at 94°C, 35 cycles of amplification (30" denaturation at 94°C, 30" annealing at 59.5°C, and 1' elongation at 72°C), and 5' final elongation at 72°C. PCR product was then purified using Illustra™ ExoStar™, and Sanger sequencing was performed at the ABI lab of the University of Oslo. Quality assessment, trimming, and manual checking were performed in Geneious® v6.1.2. Unfortunately, our HVR sequences were of consistently low quality and thus could not be used reliably in analysis. Many double peaks were observed, possibly explained by HVR duplication (as previously observed in *Thalassarche* albatrosses[8]), together with possible instances of polynucleotide repeat number variation. The uncertainties regarding base calls and site phasing in the case of double peaks made it impossible to extract any reliable data from these sequences.

We also re-analysed the data from Younger and colleagues[7] (GenBank accession numbers KP644787-KP645015 and KP640645-KP640873). Considering no reliable and controlled model can account for diploid sites in mitochondrial DNA, we masked all ambiguous sites from analysis. We also considered that the higher reported similarity between Ross Sea mtDNA samples may be biased by the fact that, after masking ambiguous sites, two haplotypes are overly represented compared to the average haplotype diversity. Indeed, when concatenating HVR and

Cytochrome-*b* sequences for each individual, and putting aside the two over-represented sequences, each mitochondrial haplotype is present in an average 1.4 ±0.9 copies per colony. The two over-represented haplotypes, on the other hand, are found in 14 samples from Cape Washington, and 15 from Cape Crozier. Not surprisingly, however, these samples are the only "shed feathers [...] collected from the Ross Sea between 2010 and 2012 [...] at least 10 m apart to minimize sampling the same bird" as opposed to blood samples for the other locations. It indeed may seem rather likely that the precaution was not sufficient, and that the same birds were sampled multiple times, as it is common for a moulting penguin to shed feathers on a great surface. Once mis-called bases are masked, and potentially pseudo-replicate samples are removed, the resulting pattern does not exhibit the clear bipartite organisation found by Younger and colleagues[7], but rather a gradual differentiation, in keeping with the general pattern we observe in genomic SNP data (Supplementary Fig. 5B). As a side note, the over-representation of pseudo-replicate sequences in the Ross sea region, by violating the random-sampling assumption of coalescent reconstructions, may also account for the differences in past demographic trends inferences found by the authors (see Figure 2 in Younger *et al.*[7]).

Cytochrome-*b* sequences, on the other hand, showed a standard level of variation in our dataset, as assessed in DnaSP[9] (10 haplotypes, gene diversity = 0.634, nucleotide diversity = 0.003, Fu's Fs = -0.948, Tajima's D = -0.216, non-significant). Haplotype network was built based on Fitch distances between sequences, using Fitchi[10] and a maximum-likelihood bifurcating tree built in RaxML[11]. In keeping with the results of Younger and colleagues[7], Cytochrome-*b* sequences do not reflect geographical distribution of the samples in any way (Supplementary Fig. 5A).

The particular case of the Ross Sea area may require further analysis, as mitochondrial HVR alone seems too unreliably sequenced to provide positive information. The available data only support the extension of a low-level isolation-by-distance model to the whole continent, in keeping with our observations on genome-wide polymorphism. However, the intensity of the gene flow between the Ross Sea region and the rest of the continent has little impact on our ability to model immigration rates at the colony level. Indeed, the origin of the immigration flux is less relevant than its intensity if we are to accurately model population dynamics from colony-level data.

Supplementary References

1. Berthold, P. Genetic control of migratory behaviour in birds. *Trends Ecol. Evol.* **6,** 254-257 (1991).
2. Bost, C. A., Thiebot, J. B., Pinaud, D., Cherel, Y. & Trathan, P. N. Where do penguins go during the inter-breeding period? Using geolocation to track the winter dispersion of the macaroni penguin. *Biol. Lett.* **5,** 473-476 (2009).
3. Howard, W. E. Innate and environmental dispersal of individual vertebrates. *Am. Midl. Nat.* **63,** 152-161 (1960).
4. Dingle, H. & Drake, V. A. What is migration. *Bioscience* **57,** 113-121 (2007).
5. Beerli, P. & Palczewski, M. Unified framework to evaluate panmixia and migration direction among multiple sampling locations. *Genetics* **185,** 313-326 (2010).
6. Beerli, P. Effect of unsampled populations on the estimation of population sizes and migration rates between sampled populations. *Mol. Ecol.* **13,** 827-836 (2004).
7. Younger, J. L. *et al.* Too much of a good thing: sea ice extent may have forced emperor penguins into refugia during the last glacial maximum. *Glob. Chang. Biol.* **21,** 2215-2226 (2015).
8. Abbott, C. L., Double, M. C., Trueman, J. W. H., Robinson, A. & Cockburn, A. An unusual source of apparent mitochondrial heteroplasmy: duplicate mitochondrial control regions in Thalassarche albatrosses. *Mol. Ecol.* **14,** 3605-3613 (2005).
9. Rozas, J. & Rozas, R. DnaSP version 3: an integrated program for molecular population genetics and molecular evolution analysis. *Bioinformatics* **15,** 174-175 (1999).
10. Matschiner, M. Fitchi: Haplotype genealogies based on Fitch distances. http://www.evoinformatics.eu/fitchi (2015).
11. Stamatakis, A. RAxML version 8: a tool for phylogenetic analysis and post-analysis of large phylogenies. *Bioinformatics* **30,** 1312-1313 (2014).
